# Hypotonia and intellectual disability without dysmorphic features in a patient with *PIGN*-related disease

**DOI:** 10.1186/s12881-017-0481-9

**Published:** 2017-11-02

**Authors:** Isabelle Thiffault, Britton Zuccarelli, Holly Welsh, Xuan Yuan, Emily Farrow, Lee Zellmer, Neil Miller, Sarah Soden, Ahmed Abdelmoity, Robert A. Brodsky, Carol Saunders

**Affiliations:** 1 Center for Pediatric Genomic Medicine, Children’s Mercy Hospital, 2420 Pershing Road, Kansas City, MO 64108 USA; 2 Department of Pathology and Laboratory Medicine, Children’s Mercy Hospitals, Kansas City, MO USA; 3 University of Missouri–Kansas City School of Medicine, Kansas City, MO USA; 4 Department of Pediatrics, Children’s Mercy Hospitals, Kansas City, MO USA; 5 Johns Hopkins Division of Hematology, Baltimore, MD USA

**Keywords:** *PIGN*, Developmental disorders, Intellectual disability, GPI deficiency, Seizures

## Abstract

**Background:**

Defects in the human glycosylphosphatidylinositol anchor biosynthetic pathway are associated with inherited glycosylphosphatidylinositol (GPI)-deficiencies characterized by a broad range of clinical phenotypes including multiple congenital anomalies, dysmorphic faces, developmental delay, hypotonia, and epilepsy. Biallelic variants in *PIGN*, encoding phosphatidylinositol-glycan biosynthesis class N have been recently associated with multiple congenital anomalies hypotonia seizure syndrome.

**Case presentation:**

Our patient is a 2 year old male with hypotonia, global developmental delay, and focal epilepsy. Trio whole-exome sequencing revealed heterozygous variants in *PIGN*, c.181G > T (p.Glu61*) and c.284G > A (p.Arg95Gln). Analysis of FLAER and anti-CD59 by flow-cytometry demonstrated a shift in this patient’s granulocytes, confirming a glycosylphosphatidylinositol-biosynthesis defect, consistent with *PIGN*-related disease.

**Conclusions:**

To date, a total of 18 patients have been reported, all but 2 of whom have congenital anomalies and/or obvious dysmorphic features. Our patient has no significant dysmorphic features or multiple congenital anomalies, which is consistent with recent reports linking non-truncating variants with a milder phenotype, highlighting the importance of functional studies in interpreting sequence variants.

**Electronic supplementary material:**

The online version of this article (10.1186/s12881-017-0481-9) contains supplementary material, which is available to authorized users.

## Background

Biallelic variants in glycosylphosphatidylinositol (GPI)-anchor synthesis pathway genes are responsible for GPI deficiency disorders, associated with broad clinical features including intellectual disability, seizures, and diverse congenital anomalies. At least 150 different human proteins are anchored by the glycolipid GPI to the outer plasma membrane [1]. GPI-anchoring is a multistep process which includes; 1) synthesis of the GPI precursor molecules in the endoplasmic reticulum (ER), 2) transfer to the carboxy-terminus of the protein, and 3) remodeling of the GPI-protein complex in the ER and Golgi [2]. Complete GPI deficiency is expected to cause embryonic lethality because GPI proteins with important roles in embryogenesis require GPI anchoring for expression on the cell surface [3]. In support of this, all reported cases of GPI deficiency have been due to hypomorphic variants. At least 26 genes involved in the biosynthesis and remodelling of GPI anchored proteins have been described [4, 5], with germline pathogenic variants in 13 have been so far associated with a variety of human disorders. Whole-exome sequencing (WES) permits the simultaneous interrogation of all inborn GPI deficiency gene sequences including *PIGV* [6], *PIGN* [7], *PIGL* [8], *PIGA* [9], *PIGO* [10], *PGAP2* [11], and *PIGT* [12], as well as many other neurological disorders on the differential. Biallelic variants in *PIGN*, have been recently associated with Multiple Congenital Anomalies-Hypotonia-Seizures syndrome 1 (MCAHS1; OMIM #614080) is an autosomal recessive disorder, and so is Fryns syndrome. Both disorders have actually been reported to be associated to PIGN-deficiency, similarly MCAHS2 (OMIM #300868) is linked to *PIGA* defects [7], and MCAHS3 (OMIM #615398) to *PIGT* defects [12, 7, 11, 13–21]. To date, a total of 18 patients have been reported, all but 2 of whom have congenital anomalies and/or obvious dysmorphic features [7, 11, 13–21]. Here, we report a patient with *PIGN*-related GPI deficiency and no congenital anomalies or obvious dysmorphic features.

## Case presentation

We present a 2 year old male (CMH1157) with hypotonia, global developmental delay and focal epilepsy. He was born at 38-weeks gestation following an uncomplicated pregnancy. The patient’s mother was a 33 year old gravida 2 para 2 female. There was no exposure to alcohol, tobacco, or drugs. Delivery was via spontaneous vaginal delivery. His growth parameters at birth included a weight of 3.46 kg (90% for gestational age) and length of 47 cm (30% for gestational age). At 2 months of age his pediatrician raised concern for developmental delay because he had not yet developed a social smile, did not yet fix nor track, and had poor head control. He began having involuntary movements at 3 months of age that were initially diagnosed as Sandifer syndrome. These events were ultimately classified as partial complex seizures, which were controlled with levetiracetam and topiramate. The family history was notable for a maternal grandmother who died from complications related to what was described as a rapid-onset ataxia followed by the development of aphasia and seizures. No genetic diagnostic evaluation was ever performed for this individual. The patient’s parents and 3 year old sister were otherwise healthy.

**Fig. 1:**
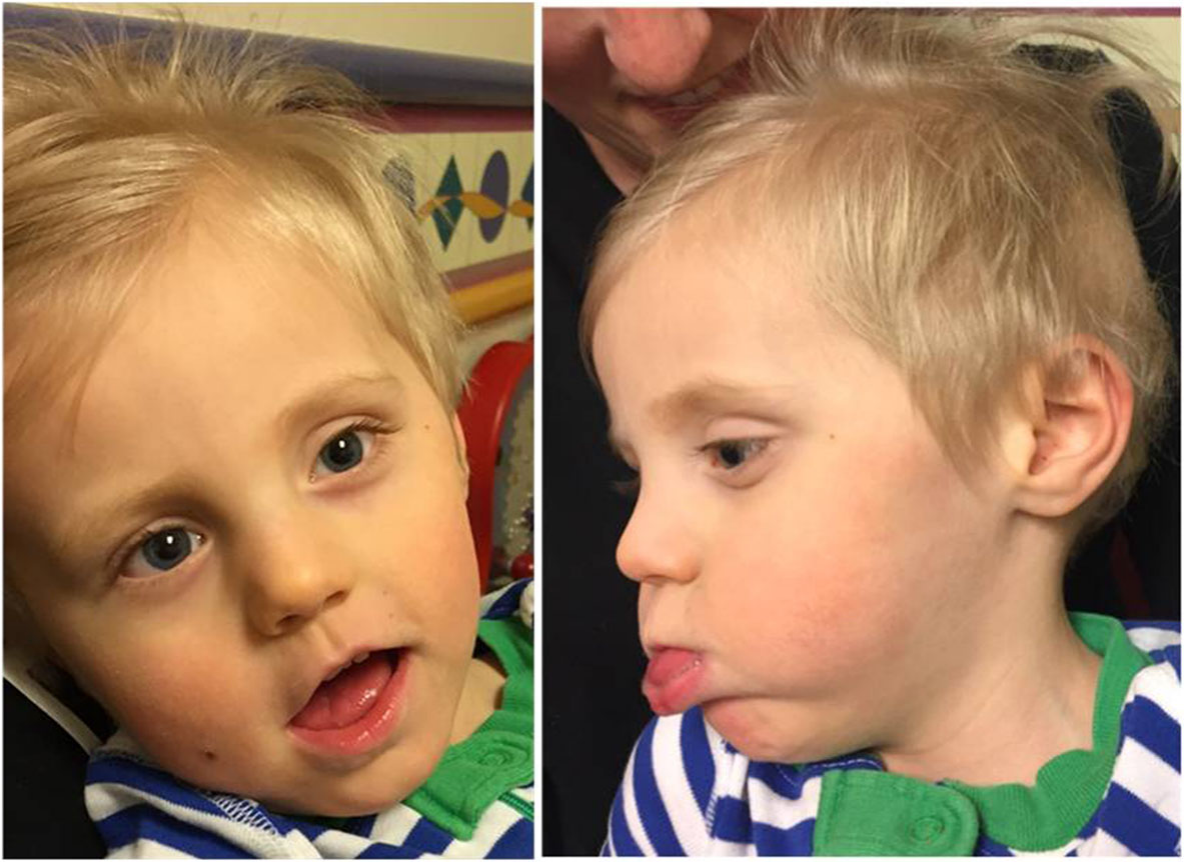
Patient CMH1157 at nearly 3 years of age, displaying marked hypotonia without dysmorphic features

During his initial neurodiagnostic evaluation, no source of provocation for seizure was identified. Laboratory studies including lactate, pyruvate, creatine kinase, serum amino acids, plasma acylcarnitines, and urine organic acids were unremarkable. Karyotype and microarray were unremarkable. His electroencephalogram revealed right centro-temporal focal slowing. A brain magnetic resonance imaging study revealed an incidental left middle cranial fossa arachnoid cyst and mildly enlarged subarachnoid spaces but was otherwise unremarkable and did not identify a potential seizure focus. Negative molecular studies included testing for Prader-Willi and Fragile X syndromes, and sequencing for mitochondrial tRNA mutations. A panel of 51 genes associated with epilepsy performed in 2014, revealed a heterozygous variant of unknown significance (c.68C > A, p.Ala23Glu) in the *SCN1A* gene. Forty-five hours of continuous monitoring for spells characterization at 5 months of age confirmed their epileptic nature, with right hemispheric discharges. He was continued on levetiracetam at 45 mg/kg/day and started on adjunctive topiramate at 7 mg/kg/day after which he achieved seizure-freedom. At 22 months of age he remained profoundly delayed, unable to hold his head up, roll over, or sit, with no babbling or other verbal skills. Further evaluation was performed for a suspected underlying genetic condition due to his history of poor growth (weight < 2nd percentile, length < 10th percentile), relative macrocephaly / brachyephaly (head circumference 85th percentile), focal epilepsy, hypotonia, developmental delays, cortical visual impairment, and gastrointestinal difficulties. Although he does not have striking dysmorphia, a physical exam at 8 months of age noted a short nose, tall palate, and hypoplasia of the 4th and 5th distal phalanges (Fig. 1).

WES was performed on the patient and his healthy parents, following informed consent and CARE guidelines. Genomic DNA was extracted from peripheral blood mononuclear cells using a Chemagen MSM1 robot (Perkin Elmer) and prepared for sequencing using the Nextera Rapid Capture Exome kit. Sequencing was completed on an Illumina HiSeq 2500 instrument. Approximately 7.62 Gb of 2 × 125 sequenced paired end reads were obtained, for a mean of 78× average coverage. Bidirectional sequence was assembled, aligned to reference gene sequences based on human genome build GRCh37/UCSC hg19, and analyzed using custom-developed software, RUNES and VIKING [22], variants were filtered to 1% minor allele frequency in our variant database, then prioritized by the American College of Medical Genetics (ACMG) categorization [23], OMIM identity and phenotypic assessment. Alignments were viewed using Integrative Genomic Viewer software version 2.3.8 (IGV; Broad Institute, Cambridge, MA, USA). Because the disorder was thought to be either recessive or due to a de novo mutation, the initial analysis targeted homozygous or compound heterozygous variants with an allele frequency less than 1% in our local database of >5000 samples, or variants unique to the patient.

After filtering for OMIM phenotypes consistent with this patient’s clinical presentation, we retained only two genes, *SCN1A* and *PIGN*. The paternally inherited *SCN1A* variant previously identified through clinical testing, p.Ala23Glu (c.68C > A), was confirmed by WES. In addition, this patient was found to be *compound heterozygous* in the *PIGN* gene for two variants. The first, c.181G > T (p.Glu61*), was a maternally-inherited nonsense variant in exon 4 of the gene. Though this variant had not been previously reported, it is of the type expected to be pathogenic, the mRNA produced will likely be targeted for nonsense mediated decay, resulting in a degraded product. This variant is found in only one heterozygous individual in the ExAC database so is extremely rare in the general population (Additional file 1: Table S1). The second variant, c.284G > A (p.Arg95Gln), results in the substitution of a highly conserved arginine to glutamine in exon 5, a change that is predicted to be deleterious by in silico programs. This paternally-inherited variant is also very rare in the general population, found in only 2 controls in the ExAC database (Additional file 1: Table S1). In the absence of additional evidence of pathogenicity, this missense variant was interpreted as a variant of unknown significance (Additional file 1: Table S1). The genotypes of the proband and parents were confirmed clinically with Sanger sequencing.

**Fig. 2:**
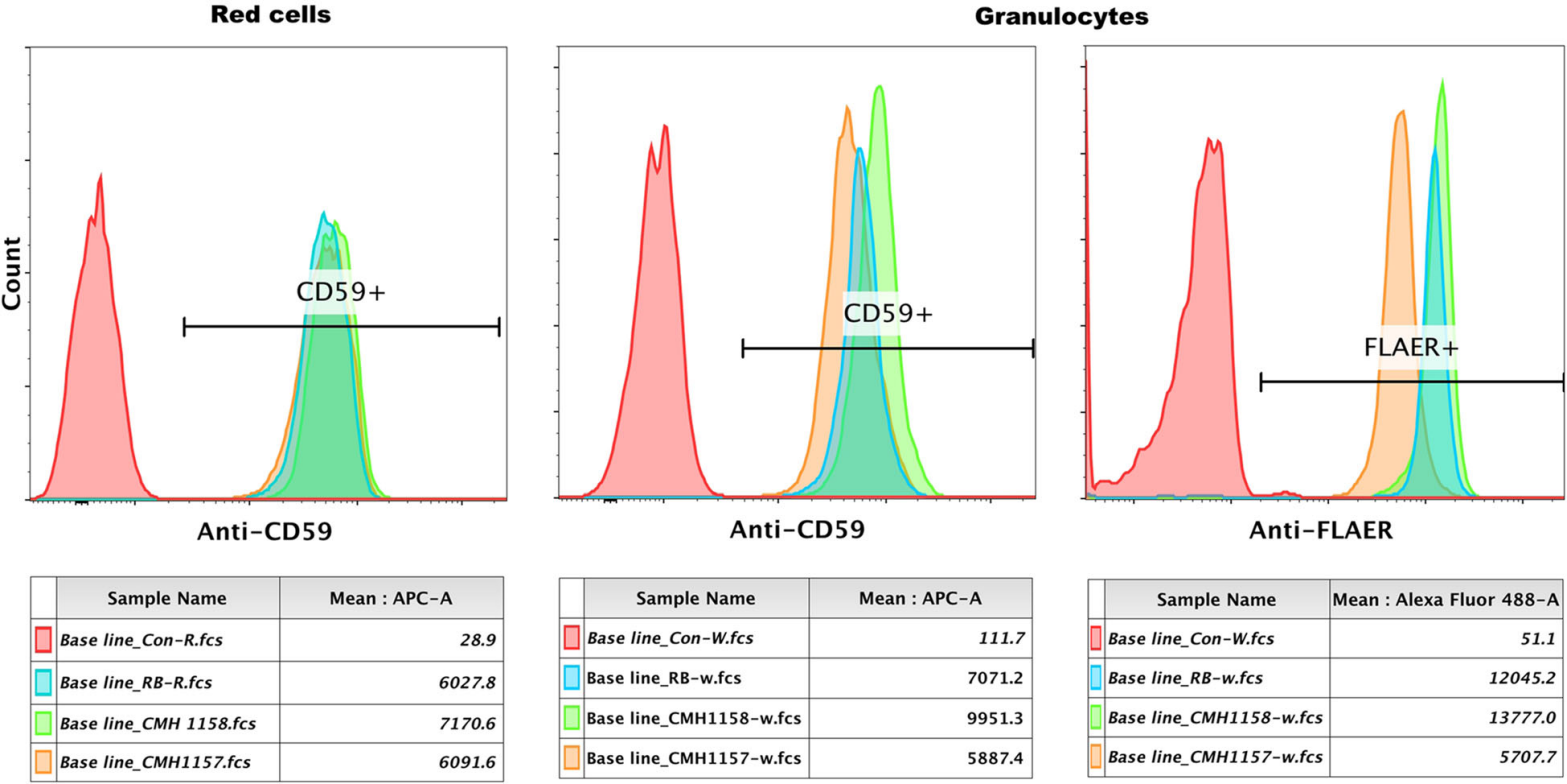
Cell surface display of GPI-anchored proteins on red cells and granulocytes analyzed by flow cytometry. Red cells were stained with mouse anti-human CD59. Red cells from the patient (1157), his mother (1158) and a healthy control RB displayed similar amounts of CD59. However, patient granulocytes (1157) showed a half log reduction in fluorescence intensity following staining with anti-human CD59 and FLAER compared to the carrier mother (1158) and a healthy male control (RB)

Though suspected to be related to the patient’s phenotype, the uncertainty surrounding the pathogenicity of the missense variant called for functional studies to prove causality before interpreting this genotype as pathogenic. In order to examine the effect of the genotype, especially the p.Arg95Gln variant on the function of PIGN, the surface expression of GPI-anchored proteins on granulocytes were analyzed by flow cytometry analysis (FACS). Granulocytes from the patient and reference controls were gated after staining with mouse anti-human CD59 and FLAER [24] to allow us to perform the FACS analysis only on blood granulocytes. Such studies (Fig. 2) demonstrated a shift in anti-CD59 and FLAER staining in this patient’s granulocytes, indicating a GPI defect consistent with *PIGN*-related disease. Taken together with the fact that this variant was found *in trans* with a pathogenic variant, p.Arg95Gln was classified as likely pathogenic and the genotype interpreted to be diagnostic for *PIGN*-related disease.

## Discussion and conclusions

Of the 26 genes involved in the GPI pathway, four are required for remodeling of GPI after attachment to proteins. In addition, 22 so-called “PIG genes” are required for synthesis and protein attachment of GPI, with *PIGN* belonging to the latter group [2, 4, 5]. Variants in *PIGN* are associated with a variable phenotype including epilepsy, hypotonia, global developmental delay, gastroesophageal reflux, and congenital anomalies of the hands, feet, heart, gastrointestinal system, genitourinary system, and brain [7, 13–15, 20, 21]. A recent genotype-phenotype correlation has been suggested, where congenital anomalies are found only in association with biallelic truncating variants [15]; our patient further supports this.

This case is one of many demonstrating the utility of WES in diagnosing neurodevelopmental disorders in the context of clinical testing. Targeted panels exist for many disease states, and have benefits including less data to interpret, minimizing incidental findings, and are generally less expensive than WES or WGS. However, the diagnostic yield of this approach is <10% (cjs, unpublished data), well below of that of WES/WGS, which ranges from 25 to 50%. Panel testing often necessitates more than one test, and serial testing of additional genes or panels quickly surpasses the expense of WES/WGS [22]. Such panels are more economical only if the relevant gene is covered, and the extreme variability of the content different laboratories offer for the same condition makes it difficult for clinicians to manage the gene lists for such nonspecific symptoms such as intellectual disability. The rapid rate of new gene discovery makes it difficult for laboratories to incorporate relevant targets to panels, even if the difference in the procedure is simply the bioinformatic unmasking of genes. For example, *PIGN*, which was first reported to have a disease association in May of 2014, is represented on only 2 of 47 Epilepsy panels and 2 of 24 Autism/Intellectual disability panels listed in NextGxDx. The comparison of such gene lists is difficult for clinicians and the curation is onerous for the clinical laboratory to manage. Clinical WES/WGS removes the guesswork related to gene inclusion, since all genes relevant to the patient’s phenotype are queried in the analysis process. In the current case, by using WES in a young child with hypotonia, seizures, a diagnosis of *PIGN*-related disease was made.

The clinical severity of the cases reported to date seems to correlate with the predicted functional severity of the pathogenic variants seen in *PIGN*. The male we described here further supports the genotype-phenotype assertions*.* He has marked phenotypic overlap with the previously reported cases. More recently, Fryns syndrome can be caused by recessive mutations in *PIGN*, providing further evidence for genetic heterogeneity [16, 17]. The patient we report and two recent published reports [13, 15] suggest that major congenital anomalies are not a core feature of PIGN-related disorders and are associated only in the presence of two truncating variants. Evaluation for pathogenic variants in genes involved the GPI-anchor synthesis pathway, causing PIG-associated epilepsy/multiple congenital anomalies-hypotonia-seizures syndrome, should be considered in patients of all ethnicities with epilepsy, with or without additional features. The increasing number of phenotypes associated with pathogenic variants (coding and non-coding) in the GPI pathway suggests that expansion of genotype-phenotype correlations related to GPI pathophysiology still requires further investigations.

## Additional file

Additional file 1: Table S1. Characteristic of variants reported in patient CMH1157. (DOCX 34 kb)

## Abbreviations

ACMG: American College of Medical Genetics
DNA: Deoxyribonucleic acid
ER: Endoplasmic reticulum
GPI: Glycosylphosphatidylinositol
IGV: Integrative Genomics Viewer
OMIM: Online Mendelian Inheritance in Man
WES: Whole-exome sequencing
WGS: Whole-genome sequencing

## References

[1] Fujita M, Kinoshita T (2012). GPI-anchor remodeling: potential functions of GPI-anchors in intracellular trafficking and membrane dynamics. Biochim Biophys Acta.

[2] Ng BG, Freeze HH (2015). Human genetic disorders involving glycosylphosphatidylinositol (GPI) anchors and glycosphingolipids (GSL). J Inherit Metab Dis.

[3] Yuan X, Braunstein EM, Ye Z, Liu CF, Chen G, Zou J, Cheng L, Brodsky RA (2013). Generation of glycosylphosphatidylinositol anchor protein-deficient blood cells from human induced pluripotent stem cells. Stem Cells Transl Med.

[4] Kinoshita T (2014). Enzymatic mechanism of GPI anchor attachment clarified. Cell Cycle.

[5] Kinoshita T (2014). Biochemistry of glycosylphosphatidylinositol (GPI) anchored proteins. Seikagaku J Japanese Biochem Soc.

[6] Krawitz PM, Schweiger MR, Rodelsperger C, Marcelis C, Kolsch U, Meisel C, Stephani F, Kinoshita T, Murakami Y, Bauer S (2010). Identity-by-descent filtering of exome sequence data identifies PIGV mutations in hyperphosphatasia mental retardation syndrome. Nat Genet.

[7] Maydan G, Noyman I, Har-Zahav A, Neriah ZB, Pasmanik-Chor M, Yeheskel A, Albin-Kaplanski A, Maya I, Magal N, Birk E (2011). Multiple congenital anomalies-hypotonia-seizures syndrome is caused by a mutation in PIGN. J Med Genet.

[8] Ng BG, Hackmann K, Jones MA, Eroshkin AM, He P, Wiliams R, Bhide S, Cantagrel V, Gleeson JG, Paller AS (2012). Mutations in the glycosylphosphatidylinositol gene PIGL cause CHIME syndrome. Am J Hum Genet.

[9] Johnston JJ, Gropman AL, Sapp JC, Teer JK, Martin JM, Liu CF, Yuan X, Ye Z, Cheng L, Brodsky RA (2012). The phenotype of a germline mutation in PIGA: the gene somatically mutated in paroxysmal nocturnal hemoglobinuria. Am J Hum Genet.

[10] Krawitz PM, Murakami Y, Hecht J, Kruger U, Holder SE, Mortier GR, Delle Chiaie B, De Baere E, Thompson MD, Roscioli T (2012). Mutations in PIGO, a member of the GPI-anchor-synthesis pathway, cause hyperphosphatasia with mental retardation. Am J Hum Genet.

[11] Hansen L, Tawamie H, Murakami Y, Mang Y, ur Rehman S, Buchert R, Schaffer S, Muhammad S, Bak M, Nothen MM (2013). Hypomorphic mutations in PGAP2, encoding a GPI-anchor-remodeling protein, cause autosomal-recessive intellectual disability. Am J Hum Genet.

[12] Kvarnung M, Nilsson D, Lindstrand A, Korenke GC, Chiang SC, Blennow E, Bergmann M, Stodberg T, Makitie O, Anderlid BM (2013). A novel intellectual disability syndrome caused by GPI anchor deficiency due to homozygous mutations in PIGT. J Med Genet.

[13] Nakagawa T, Taniguchi-Ikeda M, Murakami Y, Nakamura S, Motooka D, Emoto T, Satake W, Nishiyama M, Toyoshima D, Morisada N (2016). A novel PIGN mutation and prenatal diagnosis of inherited glycosylphosphatidylinositol deficiency. Am J Med Genet A.

[14] Khayat M, Tilghman JM, Chervinsky I, Zalman L, Chakravarti A, Shalev SA (2016). A PIGN mutation responsible for multiple congenital anomalies-hypotonia-seizures syndrome 1 (MCAHS1) in an Israeli-Arab family. Am J Med Genet A.

[15] Fleming L, Lemmon M, Beck N, Johnson M, Mu W, Murdock D, Bodurtha J, Hoover-Fong J, Cohn R, Bosemani T (2016). Genotype-phenotype correlation of congenital anomalies in multiple congenital anomalies hypotonia seizures syndrome (MCAHS1)/PIGN-related epilepsy. Am J Med Genet A.

[16] Thompson MD, Cole DE (2016). Recessive PIGN mutations in Fryns syndrome: evidence for genetic heterogeneity. Hum Mutat.

[17] McInerney-Leo AM, Harris JE, Gattas M, Peach EE, Sinnott S, Dudding-Byth T, Rajagopalan S, Barnett CP, Anderson LK, Wheeler L (2016). Fryns syndrome associated with recessive mutations in PIGN in two separate families. Hum Mutat.

[18] Jezela-Stanek A, Ciara E, Piekutowska-Abramczuk D, Trubicka J, Jurkiewicz E, Rokicki D, Mierzewska H, Spychalska J, Uhrynowska M, Szwarc-Bronikowska M (2016). Congenital disorder of glycosylphosphatidylinositol (GPI)-anchor biosynthesis--the phenotype of two patients with novel mutations in the PIGN and PGAP2 genes. Eur J Paediatr Neurol.

[19] Krawitz PM, Murakami Y, Riess A, Hietala M, Kruger U, Zhu N, Kinoshita T, Mundlos S, Hecht J, Robinson PN (2013). PGAP2 mutations, affecting the GPI-anchor-synthesis pathway, cause hyperphosphatasia with mental retardation syndrome. Am J Hum Genet.

[20] Ohba C, Okamoto N, Murakami Y, Suzuki Y, Tsurusaki Y, Nakashima M, Miyake N, Tanaka F, Kinoshita T, Matsumoto N (2014). PIGN mutations cause congenital anomalies, developmental delay, hypotonia, epilepsy, and progressive cerebellar atrophy. Neurogenetics.

[21] Brady PD, Moerman P, De Catte L, Deprest J, Devriendt K, Vermeesch JR (2014). Exome sequencing identifies a recessive PIGN splice site mutation as a cause of syndromic congenital diaphragmatic hernia. Eur J Med Genet.

[22] Soden SE, Saunders CJ, Willig LK, Farrow EG, Smith LD, Petrikin JE, LePichon JB, Miller NA, Thiffault I, Dinwiddie DL (2014). Effectiveness of exome and genome sequencing guided by acuity of illness for diagnosis of neurodevelopmental disorders. Sci Transl Med.

[23] Richards S, Aziz N, Bale S, Bick D, Das S, Gastier-Foster J, Grody WW, Hegde M, Lyon E, Spector E (2015). Standards and guidelines for the interpretation of sequence variants: a joint consensus recommendation of the American College of Medical Genetics and Genomics and the Association for Molecular Pathology. Genet Med.

[24] Brodsky RA, Mukhina GL, Li S, Nelson KL, Chiurazzi PL, Buckley JT, Borowitz MJ (2000). Improved detection and characterization of paroxysmal nocturnal hemoglobinuria using fluorescent aerolysin. Am J Clin Pathol.

