# Supplementary material for: Hypotonia and intellectual disability without dysmorphic features in a patient with PIGN-related disease
Source: BMC Med Genet. 2017 Nov 2;18:124. doi: 10.1186/s12881-017-0481-9 (PMC5668960; doi:10.1186/s12881-017-0481-9)
Supplement: Additional file 1: Table S1. — Characteristic of variants reported in patient CMH1157. (DOCX 34 kb) [file 12881_2017_481_MOESM1_ESM.docx]

**Table S1 Characteristic of Variants detected and reported in CMH1157**

| **Gene** | **Coding change** | **Protein change** | **SIFT** | **PolyPhen2** | **dbSNP** | [**EVS**](http://evs.gs.washington.edu/EVS/PopStatsServlet?searchBy=rsID&target=rs374704368)**(all)** | **ExAC (Non-Finnish)** | **Inheritance** |
| --- | --- | --- | --- | --- | --- | --- | --- | --- |
| [*PIGN*](https://warehouse.cmh.internal/genes/29413) | NM_176787.4: c.284G>A | NP_789744.1: p.Arg95Gln | deleterious (0.0) | Probably damaging (0.999) | rs374704368 | 0.0082 % | 2/54416 (0.0037%) | Paternally-inherited |
| [*PIGN*](https://warehouse.cmh.internal/genes/29413) | NM_176787.4:  c.181G>T | NP_789744.1: p.Glu61Ter | n.a | n.a | rs200199765 | 0.0082% | 1/66630 (0.0015%) | Maternally-inherited |
| *SCN1A* | NM_001165963.1: c.68C>A | NP_001159435.1:  p.Ala23Glu | deleterious (0.01) | Possibly  damaging (0.492) | rs139397227  RCV000188916.2  Clinvar significance = unknown/VUS | n.a | 0/ 66736 (0%); 1/8648 (0.01% in East Asians) | Paternally-inherited |

**Legend:**

Transcripts selected accordingly to HGMD (https://portal.biobase-international.com/hgmd/pro/start.php)

n.a: not applicable
